# Qubit entanglement between ring-resonator photon-pair sources on a silicon chip

**DOI:** 10.1038/ncomms8948

**Published:** 2015-08-06

**Authors:** J. W. Silverstone, R. Santagati, D. Bonneau, M. J. Strain, M. Sorel, J. L. O'Brien, M. G. Thompson

**Affiliations:** 1 Centre for Quantum Photonics, H. H. Wills Physics Laboratory and Department of Electrical and Electronic Engineering, University of Bristol, Merchant Venturers Building, Woodland Road, Bristol BS8 1UB, UK; 2 Institute of Photonics, Department of Physics, University of Strathclyde, Wolfson Centre, 106 Rottenrow East, Glasgow G4 0NW, UK; 3 School of Engineering, University of Glasgow, James Watt South Building, Glasgow G12 8QQ, UK

## Abstract

Entanglement—one of the most delicate phenomena in nature—is an essential resource for quantum information applications. Scalable photonic quantum devices must generate and control qubit entanglement on-chip, where quantum information is naturally encoded in photon path. Here we report a silicon photonic chip that uses resonant-enhanced photon-pair sources, spectral demultiplexers and reconfigurable optics to generate a path-entangled two-qubit state and analyse its entanglement. We show that ring-resonator-based spontaneous four-wave mixing photon-pair sources can be made highly indistinguishable and that their spectral correlations are small. We use on-chip frequency demultiplexers and reconfigurable optics to perform both quantum state tomography and the strict Bell-CHSH test, both of which confirm a high level of on-chip entanglement. This work demonstrates the integration of high-performance components that will be essential for building quantum devices and systems to harness photonic entanglement on the large scale.

## Abstract (web summary)

Scalable photonic devices for quantum information processing require on-chip quantum states engineering. Here the authors report the creation of entangled photon pairs on a silicon-on-insulator chip by integrating resonant photon sources, spectral demultiplexers and reconfigurable optics in a single device.

## Graphical abstract

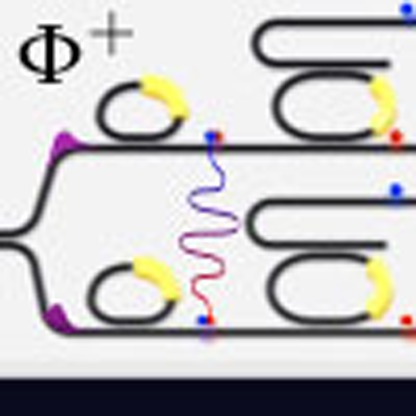

Quantum entanglement is at the heart of quantum information science: entanglement between photons and the vacuum gives security to quantum communications channels; entanglement between photons passing through a sample enables its super-resolution measurement; and entanglement between qubits provides the tremendous power behind quantum computation^1^,^2^. Qubit entanglement is regularly generated in bulk- or fibre-based quantum optical systems by directly using the intrinsic polarization correlations of the photon-pair source^3^,^4^, and on-chip using post-selected logic gates^5^,^6^. On-chip sources of photon pairs have been recently developed^7^,^8^,^9^,^10^, but rely on nonlinear processes in which all fields—pump, signal and idler—are co-polarized, both due to the increased strength of such processes and due to the difficulty of controlling polarization with integrated optics (with some exceptions^11^,^12^,^13^). Since source-based entanglement typically lies in the photonic polarization degree of freedom, on-chip sources of path-qubit entanglement have been scarce.

We present a silicon-on-insulator photonic chip operating in the central telecommunications band, which can generate and analyse the path entanglement produced by two coherently pumped photon-pair sources. As shown in Fig. 1, a pulsed pump laser is launched into two microring photon-pair sources that produce pairs in a superposition between being created in one source or the other. The device reconfigures this superposition, using on-chip demultiplexers and a waveguide crossing, into an entanglement between two photonic path qubits. Finally, these path qubits are analysed using two on-chip Mach–Zehnder interferometers (MZIs). Embedded thermo-optic modulators facilitate electro-optic control and reconfiguration of the device. The pump laser, pump-suppressing filters and detectors are all fibre integrated, off the chip. In this work, we integrate narrow-band photon sources, spectral demultiplexers and reconfigurable optics into a single device. We describe and quantify the performance of each of these functionalities, culminating with a precise estimation of the on-chip path-entangled state and a strict test of its entanglement.

## Results

### Resonant photon-pair generation

Spontaneous four-wave mixing (SFWM)^9^,^10^ is an effect of the third-order nonlinear susceptibility *χ*^(3)^ of the medium—the silicon waveguide core. We use SFWM to produce photon pairs on-chip. By convention, the two constituents of each pair are referred to as ‘signal' and ‘idler' photons, with frequencies *ν*_s_ and *ν*_i_, equally spaced on either side of the pump frequency *ν*_p_; we will refer to the higher-energy photon as the signal (that is, *ν*_s_>*ν*_p_>*ν*_i_). SFWM acts to annihilate two photons from the (degenerate) pump and create the signal and idler at new frequencies via the phenomenological Hamiltonian 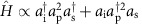. Each SFWM event conserves the energy and momentum of the input photons. In our experiment, SFWM occurs in the optical cavities formed by two microring resonators, which modify the density of states of the parametric fluorescence, and structure the spectrum of these photon pairs into bright fluorescent peaks around the cavity resonances^14^,^15^. This structure differs from the characteristic flat, broad spectrum of straight-waveguide-based sources, which is shaped by momentum conservation alone.

We pumped on resonance with the cavity at *ν*_p_, and collected signal and idler photons from adjacent cavity resonances, one free spectral range over, at *ν*_s,i_=*ν*_p_±800 GHz. The cavity linewidth was 21 GHz. Source resonances cause the highlighted dips in the transmission spectrum of Fig. 2a; the peaks in that spectrum are due to the signal–idler demultiplexers, discussed later. Our pump laser produced 10.8-ps pulses, with a 40 GHz linewidth, at a rate of 51 MHz. Since SFWM takes in two pump photons, its efficiency scales quadratically with pump power for low squeezing values. However, due to the strong two-photon absorption of silicon in the near-infrared, this quadratic scaling only holds at low power, before two-photon absorption starts to dominate^16^. In our measurements, an average pump power of 150 μW (253 mW peak) was delivered, leading to generation probabilities of 0.06 and 0.09 pairs per pulse for the two sources. System losses reduced these at-source generation rates to around 30 measured coincidences per second, with a coincidence-to-accidental ratio of around 10. Hereafter, all results are derived from net coincidence data, with accidental coincidences subtracted. An imbalance in efficiency between the top and bottom sources was somewhat compensated by the measured 54% reflectivity of the first coupler, leading to a source balance of *β*=43%.

### Quantum interference

**Figure 1:**
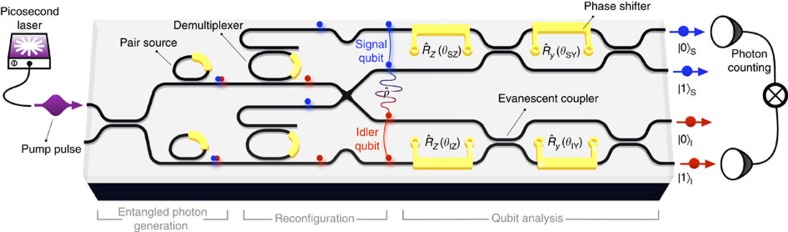
Schematic layout of the device. A picosecond pump pulse is coupled into the silicon chip where it generates a superposition of photon pairs via spontaneous four-wave mixing. This superposition is separated into signal (blue) and idler (red) path qubits, which are analysed by two MZIs. Thermo-optic phase shifters are shown in yellow. Photons at the output are separated from residual pump by fibre wavelength-division multiplexers (not shown) and collected by single-photon detectors.

Interference between photons from different sources requires those photons to be indistinguishable in all degrees of freedom, but spectral indistinguishability poses a particular challenge. We refer to this spectral indistinguishability as the overlap, *σ*, which runs from *σ*=0 for fully distinguishable photon pairs to *σ*=1 for indistinguishable ones. We explored the overlap between the two microring sources by configuring the device to interfere the signal–idler superposition on the ‘idler' interferometer of Fig. 1, which was configured as a beam splitter. We then swept one source resonance over the other (see Methods). In this way, we could observe two-photon fringes analogous to those in ref. 7, and measure changes in the quality of the quantum interference as the two sources were tuned.

**Figure 2 f2:**
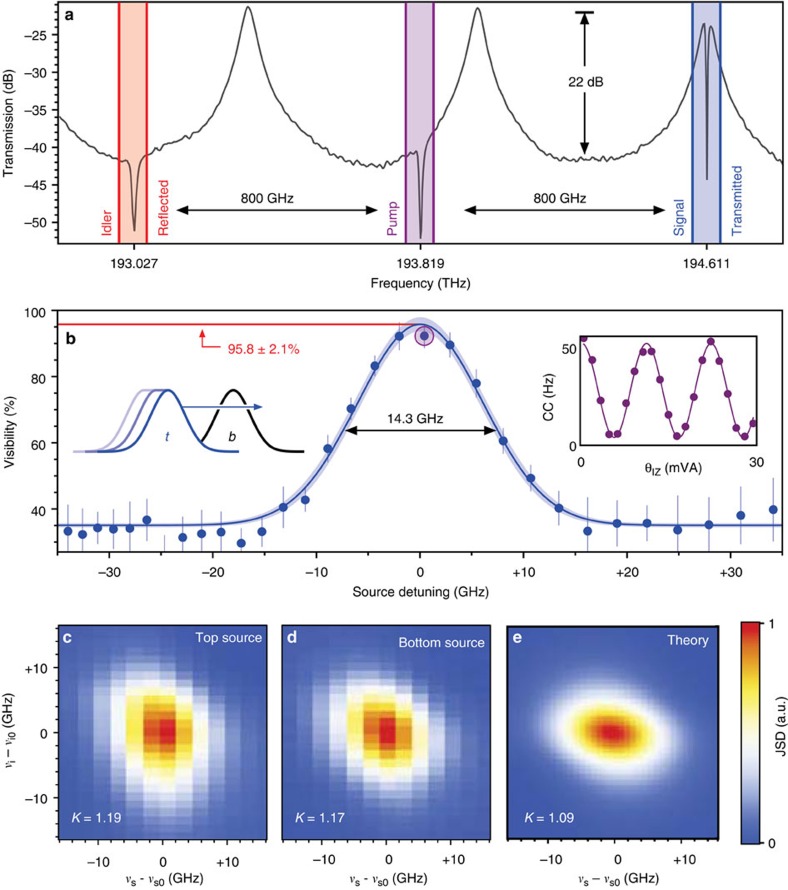
Spectral characteristics of the experiment. (**a**) Spectral layout of source (dips) and demultiplexer (peaks) resonances in the central telecommunications band. Source-free spectral range is 800 GHz, to match the 200-GHz International Telecommunication Union (ITU) grid. (**b**) Two-photon fringe visibility measured as a function of top-to-bottom source detuning, as the top resonances were scanned over the stationary bottom resonances. Left inset: diagram describing top (*t*)-source resonance sweeping across fixed bottom (*b*)-source resonance. Right inset: representative two-photon fringe corresponding to peak visibility value (circled). Residual detuned visibility is due to the interference between resonant and non-resonant pairs. Error bars represent three standard errors of each sinusoidal regression. Shaded region on fit represents one s.d. in visibility. Measured joint spectral density profiles for the top (**c**) and bottom (**d**) microring sources. (**e**) Calculated joint spectral density, based on measured linear resonator parameters.

The fringe visibility as a function of source detuning is plotted in Fig. 2b. Accounting for source imbalance, and multi-pair events, we compute the maximum visibility as 96.0% (when *σ*=1; see Supplementary Methods for details). When the sources were tuned, we observed a near-maximum peak visibility of 95.8±2.1%, corresponding to an overlap of *σ*=0.99±0.04 (Supplementary Figs 3 and 4). When the two sources were detuned completely, the visibility reached a floor of 37%. This is residual interference between broadband photon pairs produced in the non-resonant parts of the interferometer, and the single remaining resonant source. Its visibility indicates that the spectral brightness of the bus waveguide was 1% of that of the tuned microring. Since all subsequent measurements were performed through the on-chip demultiplexers, this waveguide-generated flux did not greatly affect our statistics. These data show that the two microring sources could be made indistinguishable, and exhibited brightness that dominated the background SFWM occurring in the rest of the interferometer.

The interference peak (Fig. 2b) has a Gaussian shape, indicating an inhomogeneous broadening of the SFWM emission. Its width indicates a photon linewidth of 28.6 GHz, somewhat broader than the cold cavity linewidth (21.0 GHz). Intra-cavity nonlinear effects^17^,^18^,^19^ could cause this broadening.

### Joint spectra

High-purity photon-pair sources—for heralded- or multi-photon experiments—require that, given the frequency of the signal photon, we gain minimal information about the frequency of the idler photon and vice versa—their frequency states are separable. By pumping the source cavities with spectrally broad pulses, we relax the energy and momentum requirements of the SFWM process. The emitted signal and idler photons then naturally and independently take on the structure of the cavity enhancement, which has been predicted to improve their spectral separability^20^. To quantify this separability, we measured the signal–idler joint spectral density (JSD) using the stimulated emission tomography method of ref. 21.

Measured JSD profiles are shown in Fig. 2c,d, for the top and bottom microring sources showing an overlap of *σ*=0.962. They exhibit a residual spectral entanglement (and corresponding multi-mode squeezing) with the number of modes quantified by the Schmidt number *K*, where 1/*K* would be the visibility of a triggered Hong–Ou–Mandel interference dip. We measured *K*>1.19 for the top source and *K*>1.17 for the bottom source, where *K*=1 represents perfect two-mode squeezing and spectral separability. These values represent lower bounds on *K* because our measurements only give information on the magnitude of the joint spectral amplitude, not the phase. The SFWM inside each source resonator can be understood from its bright-light transmission spectrum. This model also takes in the pump lineshape, and the waveguide dispersion and gives as output the theoretical JSD of Fig. 2e (model details are provided in Supplementary Methods). The result of this model is convolved with a Gaussian on the signal arm, to reflect the limited resolution of our spectrometer (see Methods). The measured linewidth is somewhat broader than our model's prediction, but it matches the broadening we observed in the interference measurement. In straight-waveguide source designs, spectral separability is only achievable by inserting a narrow spectral filter after the pair-generation process^7^,^11^,^22^, which necessarily reduces the source brightness. For bright heralded photon-pair sources, a naturally uncorrelated joint spectrum—like those we have shown—is desirable.

### Demultiplexing and state preparation

Each pair is produced in a superposition of being generated in the top and bottom microring sources simultaneously, since we pump with only enough power to produce one photon pair and there is a fixed phase relationship between the two microrings. This pair superposition is then converted into an entanglement between two qubits, each composed of a single signal or idler photon in one or another of two waveguide paths. The signal and idler photons are separated by on-chip demultiplexers, formed by double-bus microring resonators^23^. They exhibited a selectivity of 22 dB, a bandwidth of 35 GHz, a free spectral range of 640 GHz and a loss that was negligible compared with the system loss. They were designed to select the signal photon, while maximally rejecting the idler (see peaks in Fig. 2b). Finally, the frequency-demultiplexed waveguides are rearranged to group the signal and idler paths together. Written in the form of a density matrix and in terms of the experimental parameters *β*, *σ* and Θ, the resulting qubit-basis state is


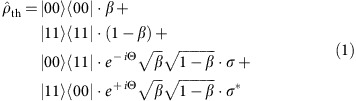


where the balance *β* describes the relative brightness of the two sources, the overlap *σ* quantifies their spectral indistinguishability and Θ accumulates the intrinsic total phase between the two qubits. We define a photon in the top (bottom) waveguide of each qubit to be a logical |0〉 (|1〉), and the first (second) qubit of each pair to be the signal (idler). For example, |00〉〈00|*β* describes both qubits having a photon in the top mode with probability *β*. Experimentally, we can control the balance *β* by adjusting the tuning of the filters (at the expense of spectral overlap); we control the overlap *σ* by tuning the two microring sources. If the flux from the two sources is balanced (*β*=1/2) and the source joint spectra overlap perfectly (*σ*=1) then 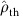 is in the family of maximally entangled Bell states. If *β*∈{0,1} then 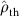 is separable; if *σ*=0 then 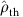 is mixed. A detailed state evolution and a short discussion on the origin of entanglement in this device can be found in the Supplementary Discussion.

### On-chip entanglement analysis

The on-chip state was manipulated and measured using integrated single-qubit analysis MZIs. These interferometers, shown in Fig. 1, implemented 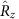 and 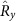 rotations by angles *θ*_SZ_, *θ*_IZ_, *θ*_SY_ and *θ*_IY_ on the signal (S) and idler (I) qubits, respectively. These rotations facilitated single-qubit measurements on the generated two-qubit state. Photons from the two qubits were counted using coincidences between two 25%-efficient avalanche photodiodes, gated on each laser pulse.

A well-known test of quantum non-locality, as well as an indicator of the entanglement present in a quantum state^24^, is based on the reformulation of Bell's original inequality due to Clauser, Horne, Shimony and Holt (CHSH)^25^. In this test, a parameter *S* is evaluated^26^, which indicates the presence of non-locality: if *S*>2, the state is non-local; if 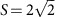 the state is maximally entangled.

**Figure 3:**
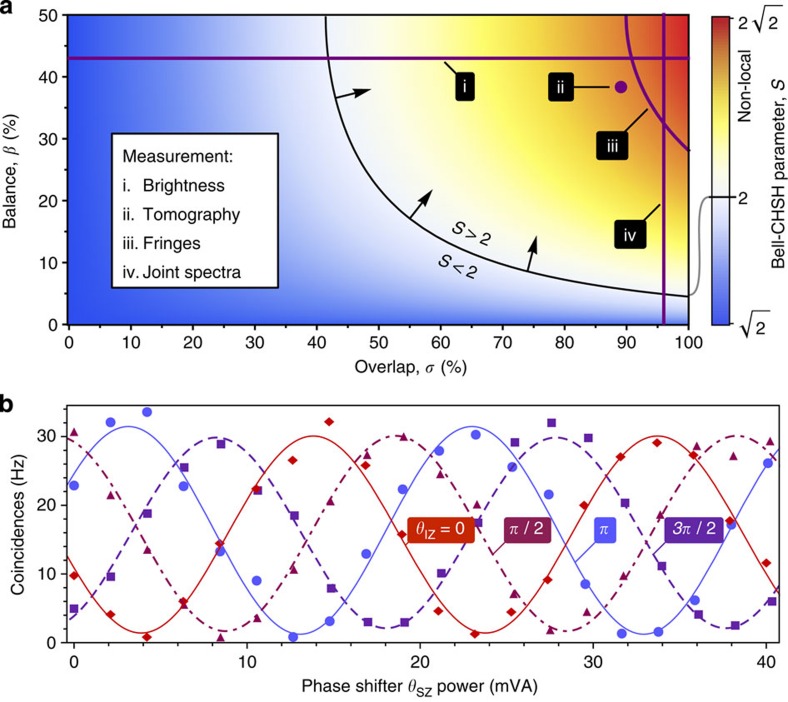
Summary of measurements in the context of Bell-CHSH inequality violation. (**a**) Map showing violation *S* as a function of source balance *β* and overlap *σ*, with listing of measurement results overlaid. When *S*>2, the measurement correlations are consistent with a non-local, entangled state. By measuring: (i) the brightness of each source, we can estimate the balance *β*; (ii) the quantum state via quantum state tomography, we can estimate both the balance *β* and the overlap *σ*; (iii) correlated fringes, we obtain a value for the violation *S*(*β*, *σ*); and (iv) the overlap between measured joint spectra gives *σ*. The measurement of *σ* in (iv) naturally excludes multi-photon contamination, while the other measurements (i–iii) necessarily include it and result in lower values of *σ* as a consequence. (**b**) Fringes generated by 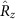 rotations on signal and idler qubits allowing a direct measurement of CHSH *S* parameter (denoted measurement (iii) in part **a**).

We can explicitly calculate the *S*, which results from 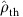 of equation (1), to quantify how the violation depends on the balance and source overlap (see Supplementary Methods):


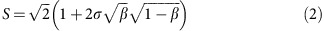


which reaches the maximum violation of 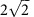 when *σ*=1 and *β*=1/2, and decreases as the overlap and balance deviate from these values. Equation (2) is plotted as a heat map in Fig. 3a showing the level of entanglement indicated by each of our measurements (i–iv).

One manifestation of the entanglement present in our on-chip state (equation (1)) is the presence of the non-local phase factor Θ. As a result of this factor, 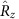 rotations applied to each qubit cannot be observed independently: each equally contributes to the total phase of the state Θ. To demonstrate this, we configured the signal and idler 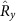 rotations to mix the two modes of each qubit (*θ*_SY_=*θ*_IY_=*π*/2), then manipulated both *θ*_SZ_ and *θ*_IZ_, and observed coincidence fringes with an entangled phase Θ=*θ*_SZ_+*θ*_IZ_ shown in Fig. 3b. These fringes have a mean visibility of 94.7±1.0% that is consistent with a strong CHSH violation of *S*=2.686±0.026. This value of *S* violates the inequality by 83% and by 26 s.d.

### Quantum state tomography

For any quantum system the total accessible information of its quantum state is encoded in its density matrix 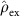. Quantum state tomography^27^ is the process of experimentally estimating 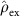 based on a series of measurements. We made an over-complete set^28^ of 36 measurements on the state using the on-chip interferometers, and used the results to estimate 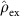. See the Methods and Supplementary Methods for details. We produced a series of on-chip states—those that were separable, mixed and entangled—by using different configurations of source and filter tuning. Manipulating both the source balance (*β*) and frequency overlap (*σ*), we observed changes in the resulting state in agreement with the predictions of equation (1).

**Figure 4:**
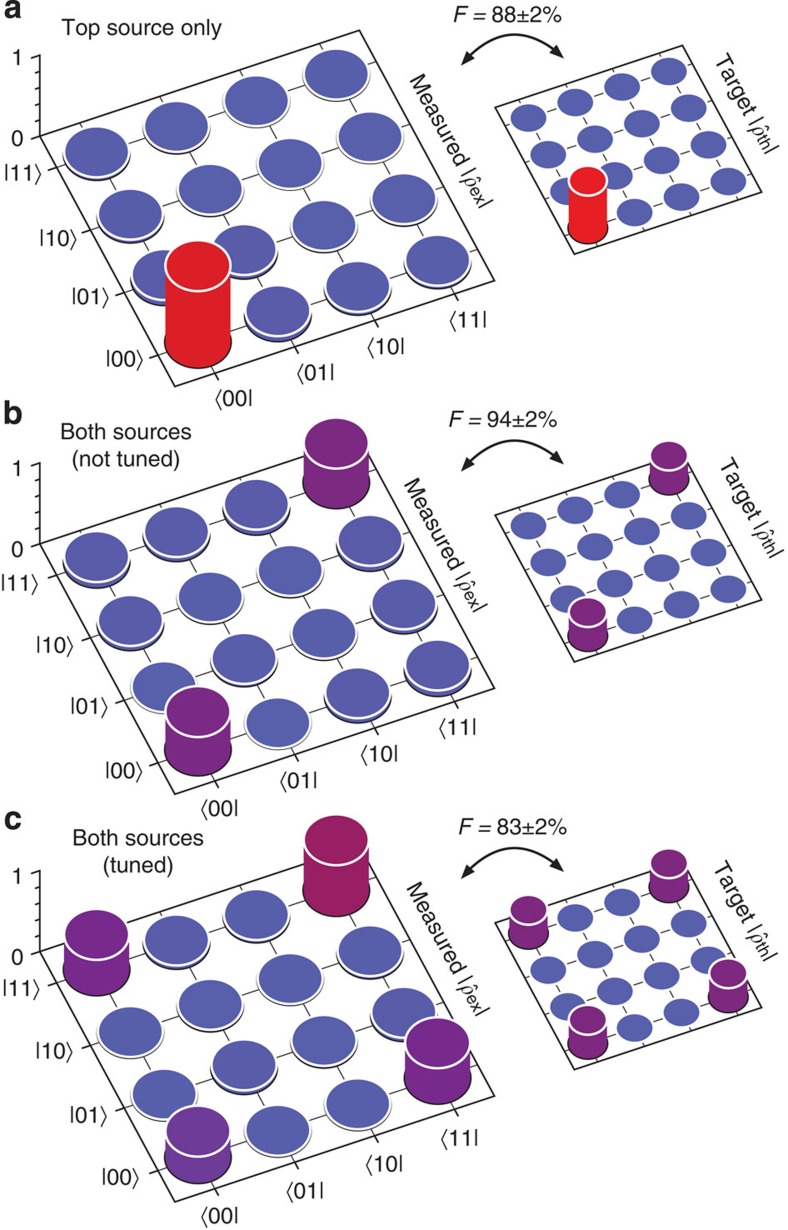
On-chip states for various device configurations, estimated using integrated analysis interferometers. Measured states are enlarged at left, with target states and corresponding fidelity (as defined in text) at right. State corresponding to (**a**) top source only (with bottom source detuned), (**b**) both sources tuned but not overlapped, showing mixed state, and (**c**) both sources tuned and overlapped, showing path-qubit entanglement.

To compare a measured state 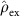 with an expected state 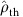, we use the fidelity *F* evaluated as


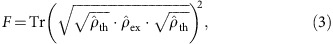


where the matrix square root is defined as 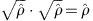. The fidelity runs from 0 to 1: *F*=1 if the states are identical, while *F*=0 if they are orthogonal. We used *F* to gauge the ability of our device to prepare particular states, comparing each measured state with the prediction of equation (1). The results for each measurement are listed in Fig. 4, beside each measured state.

In the first measurement we tuned only the top source and filter and detuned the bottom filter, effectively setting *β*=1. We estimated the state shown in Fig. 4a, which exhibits a peak in the pure |00〉 component, as expected (a similar result was obtained with the top filter detuned, with 92±1% fidelity, see Supplementary Fig. 1). Next, we tuned both filters to match each source, but did not tune the two sources to overlap, effectively setting *β*=1/2 and *σ*=0. We observed amplitude in both the |00〉 and |11〉 components, but without coherence terms (|00〉〈11| and |11〉〈00|), as shown in Fig. 4b. As predicted by equation (1) this is due to a lack of spectral overlap between photons produced in the top and bottom sources, and the resulting lack of interference at the analysis interferometers. Indeed, the estimated state is mixed with a purity of 0.49±0.01 (with 0.5 expected). Owing to the lack of coherence, we were able to use the filter lineshapes to balance the source brightness, achieving *β*=0.49. Finally, we tuned all four microrings to overlap and measured the highly entangled state of Fig. 4c, in which both sources are producing mutually coherent photons.

We evaluated the Bell-CHSH *S* parameter for the above entangled state (Fig. 4c) and found *S*=2.692±0.018. This value violates the inequality by 83% and by 38 s.d., and is in excellent agreement with our estimation based on correlated fringes (Fig. 3b).

## Discussion

We have demonstrated bright and spectrally separable photon-pair sources, phase-stable frequency-selective elements and passive and active optics integrated on a silicon chip, and used them together to generate and analyse path-qubit entanglement at optical frequencies compatible with existing telecommunications networks. We used stimulated emission tomography^21^ to provide evidence that the silicon microring source can produce spectrally uncorrelated photons—making this structure a promising candidate for future multi-pair experiments on silicon chips. Moreover, we were able to overlap two such microrings to a high degree, obtaining high-visibility quantum interference between them, despite their well-documented nonlinear dynamics^17^,^29^. We showed how the on-chip state strongly violates the Bell-CHSH inequality—a strict test of entanglement—and confirmed this experimentally in several different ways, including by on-chip quantum state tomography. That such a high degree of entanglement is generated and preserved by this device indicates the high performance of its constituent parts.

We used slow thermo-optic modulators to tune and configure the device. In our experiments, the required seconds of detector integration per reconfiguration meant this slowness posed no problem, however. Focussed efforts on thermo-optic modulation have achieved bandwidths up to 1 MHz (refs 30, 31). In any event, the thermo-optic coefficient decreases dramatically at low temperatures^32^; an entirely new approach will be required if superconducting detectors are also to be integrated^33^,^34^.

By assembling and characterizing a path-entangled Bell state on-chip, we have shown that silicon photonics, with its inherently mature and scalable manufacturing process, can prepare and manipulate delicate quantum entanglement. The narrow-band photon-pair sources and spectral demultiplexers, demonstrated here, will be useful tools for engineering future photonic quantum devices and systems.

## Methods

### Apparatus

Pump pulses were generated by a passively mode-locked fibre laser (PriTel), with a 10.8-ps duration and a 51-MHz repetition rate. These pulses were cleaned by a silica arrayed-waveguide grating with 200-GHz channel spacing, ∼120-GHz channel width and an extinction >100 dB (Opneti). Pump light was coupled onto, and signal and idler photons were coupled off of the chip via piezo-aligned lensed fibres with a 2.5-μm spot size (*e*^−2^, OZ Optics). Remaining pump was removed from signal and idler channels by two more arrayed-waveguide gratings. Signal and idler photons were detected by two InGaAs geiger-mode avalanche photodiodes with 25% nominal detection efficiency, gated to each pump pulse (ID Quantique ID210). Photon coincidences were integrated for between 10 and 30 s per measurement.

The device's eight thermo-optic phase shifters were controlled by a bank of amplified 12-bit digital-to-analog converters, controlled by computer, delivering up to 30 mW to each phase shifter. The overall temperature of the device was actively controlled using a Peltier module. Device footprint was reduced by the use of a common ground line. Electrical cross-talk due to this shared ground was mitigated using off-chip feedback.

### Device fabrication

The device was fabricated on a silicon-on-insulator wafer with a 220-nm silicon slab and a 2-μm buried oxide layer. The waveguides were 500 nm wide and were patterned using direct-write electron beam lithography into a hydrogen silsesquioxane resist layer, used as a hard mask for the reactive ion etching of the silicon slab. These structures were subsequently coated with a 900-nm silica layer. Phase shifters were based on resistive heaters, patterned atop the silica layer using a lift-off technique on a 50-nm nickel–chromium film. Electrical traces connecting the heater elements were similarly patterned in a 200-nm gold layer. An optical micrograph of the fabricated device is shown in Supplementary Fig. 5.

All waveguide–waveguide couplers were fabricated as evanescent field (directional) couplers with 300-nm gaps. Losses were minimized at waveguide crossings via tapered sections, and fibre-to-chip coupling was achieved using inverse silicon tapers embedded in 2 × 1.5-m^2^ SU8 polymer waveguides.

### Source overlap measurement

To obtain the data of Fig. 2b, we spectrally swept the top microring source resonance across the bottom one, while interfering the generated pairs on the bottom MZI (denoted 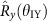 in Fig. 1). To allow both signal and idler photons to reach the bottom MZI, we detuned both filter microrings, such that they were effectively removed from the light path, and both signal and idler photons were reflected downwards. To allow interference to occur on the bottom MZI, we configured it as a simple beam splitter by setting *θ*_IY_=*π*/2. We then measured coincidences across the bottom two output ports of the device (labelled |0〉_*i*_ and |1〉_*i*_ in Fig. 1) while at the same time varying *θ*_IZ_ to form fringes. We fit these fringes sinusoidally to extract the visibility of each, and these visibility data are plotted in Fig. 2b.

### Projector calibration

The rotations 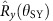, 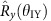, 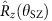 and 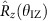 were used to analyse the states generated on-chip. To do so, we calibrated the phase–voltage relationship of each phase shifter independently. We injected laser light into the device and recorded the output intensity *I* from each interferometer as a function of the phases, obtaining *I*(*θ*_SY_,*θ*_SZ_) and *I*(*θ*_IY_,*θ*_IZ_). We fit the data with a model of the double interferometer (which included the first on-chip coupler), yielding the various coupler reflectivities and phase–voltage relationships. These data and models are plotted in Supplementary Fig. 2. Since we were unable to determine the absolute values of *θ*_SZ_ and *θ*_IZ_, we defined these phases relatively. We used the resulting models to control the on-chip phase shifters as required by each part of the experiment.

### Quantum state tomography

We used the on-chip rotations to implement an informationally over-complete^35^ set of 36 projective measurements on two qubits, to reconstruct 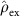. Each measurement projected each of the two qubits onto one of the six states: |0〉, |1〉, |+〉, |−〉, |+*i*〉 or |−*i*〉. We then performed a multi-dimensional search for the two-qubit state 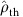, which could best explain the measurement outcomes, based on a constrained least squares estimator. The problem is defined as:


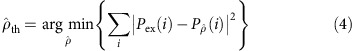


where 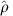 is the density matrix generated internally by the search algorithm, with the condition that it is physical: that is, hermitian, positive semi-definite and with trace one. *P*_ex_(*i*) is the *i*^th^ experimental probability estimate and 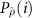 is the corresponding computed result based on the application of the *i*^th^ projector to 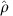.

Experimental uncertainty per count was measured using residuals from a number of coincidence fringes. This was used to estimate the uncertainty on each *P*_ex_(*i*). A Monte-Carlo method was then used to sample 500 reconstructions around the measured values *P*_ex_(*i*), and the uncertainty in each tomographic parameter (fidelity, *S*, etc.) was estimated from the distribution of these reconstructions.

### Joint spectral density measurement

In obtaining the data of Figs 2c-2d, [Fig f2], we followed closely the prescription of Liscidini *et al*.^21^ and the method of Eckstein *et al*.^36^. We tuned each ring separately and pumped them as detailed in the main text. A narrow linewidth seed laser was swept across one resonance of each ring, and the stimulated four-wave mixing was collected by a spectrometer. The seed field was provided by an amplified tuneable laser with 10 kHz linewidth (Photonetics Tunics Plus). We reduced the launched seed power until no evidence of seed-induced optical bi-stability remained. The stimulated four-wave mixing signal was collected by an optical spectrum analyser with a 6-GHz resolution (Anritsu MS9740A).

## Additional information

**How to cite this article:** Silverstone, J. W. *et al*. Qubit entanglement between ring-resonator photon-pair sources on a silicon chip. *Nat. Commun.* 6:7948 doi: 10.1038/ncomms8948 (2015).

## Supplementary Material

Supplementary Information Supplementary Figures 1-6, Supplementary Discussion and Supplementary References
