# Supplementary material for: Qubit entanglement between ring-resonator photon-pair sources on a silicon chip
Source: Nat Commun. 2015 Aug 6;6:7948. doi: 10.1038/ncomms8948 (PMC4918336; doi:10.1038/ncomms8948)
Supplement: Supplementary Information — Supplementary Figures 1-6, Supplementary Discussion and Supplementary References [file ncomms8948-s1.pdf]

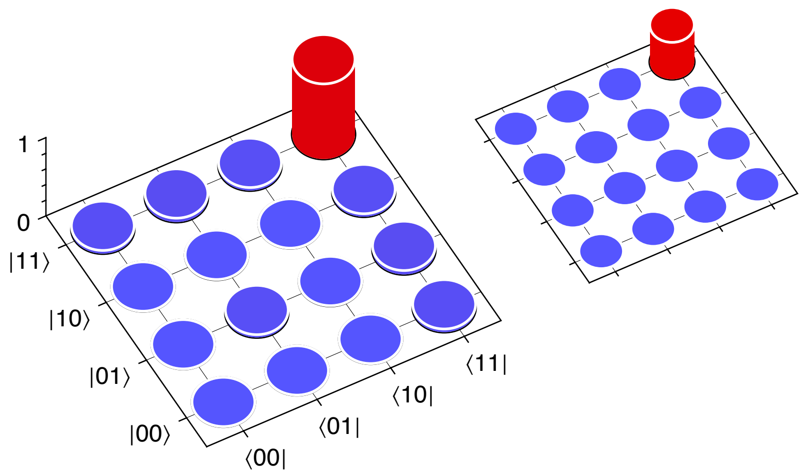

Supplementary Figure 1 Reconstructed density matrix of the separable state produced by the bottom source alone, showing a fidelity $F = 92 \pm 1\%$ compared to a pure $\hat{\rho} = |11\rangle\langle 11|$ state (shown at right).

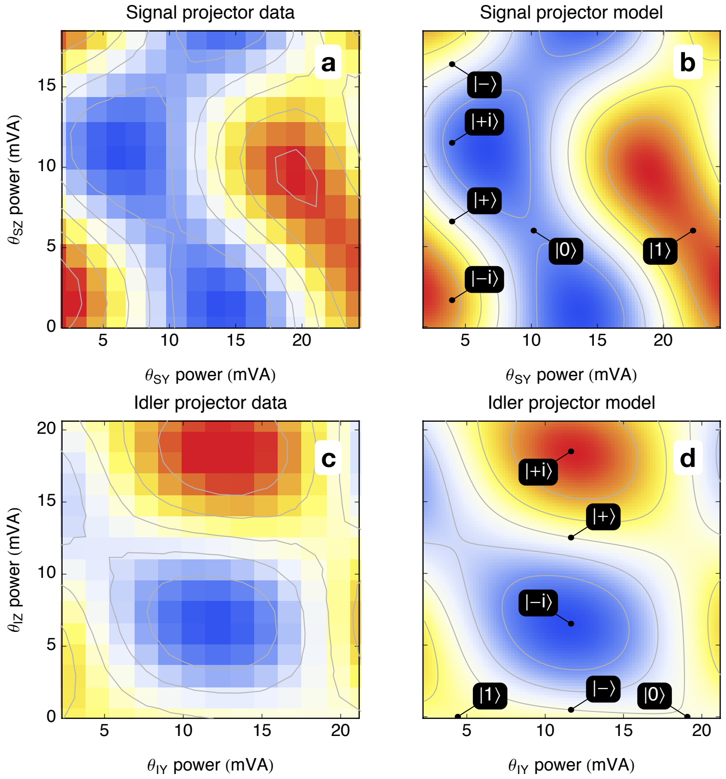

Supplementary Figure 2 Bright light calibration data (**a** and **c**) and models (**b** and **d**) for signal (**a** and **b**) and idler (**c** and **d**) qubit analysis interferometers. Optimal locations of single-qubit tomographic projectors are extracted from each model, and marked on those plots.

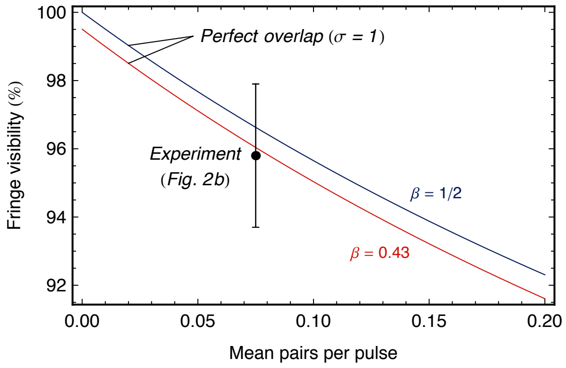

Supplementary Figure 3 Modeled maximum reverse-HOM-type fringe visibility (perfectly overlapped, $\sigma = 1$), as a function of source brightness. Curves are shown for both perfectly balanced sources ($\beta = 1/2$) and for those balanced as in this experiment ($\beta = 0.43$), with our experimental result superimposed.

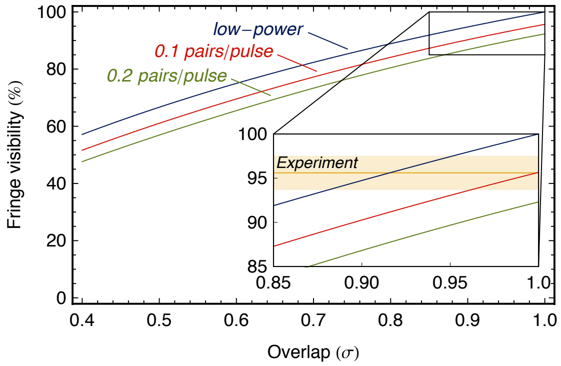

Supplementary Figure 4 Modeled visibility versus spectral overlap with balanced sources ($\beta = 1/2$), for various pair generation probabilities (and associated multi-pair probabilities). Inset shows the visibility measured in our experiment (main text Fig. 2b) together with the nearest overlap values. Substituting actual balance value ($\beta \approx 0.43$) reduces all curves by around 0.5%.

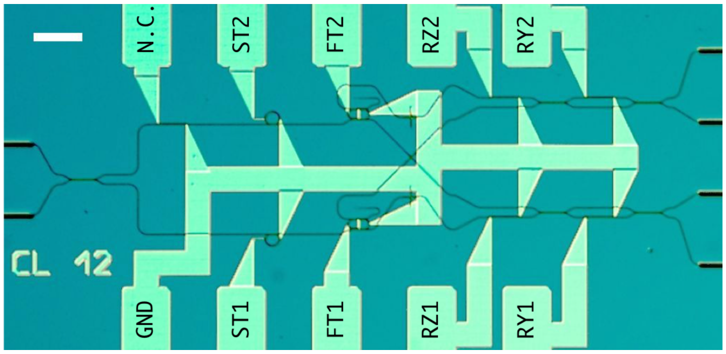

Supplementary Figure 5 Optical micrograph of the device featured in this experiment. See Fig. 1 of the main text for descriptions of individual components. A 100-μm scale bar is shown. Electrical connections are indicated: GND, ground; ST1 and ST2, source microring tuning; FT1 and FT2, filter microring tuning; RZ1 and RZ2, qubit z-rotations; RY1 and RY2, qubit y-rotations; N.C., not connected.

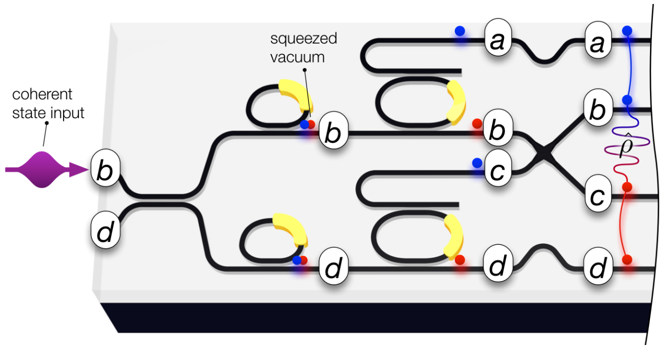

Supplementary Figure 6 Schematic of the source and state-preparation side of the device, annotated with mode labels. For use in Supplementary Discussion.

Projector	Signal qubit	Idler qubit	θ_{SZ}	θ_{SY}	θ_{IZ}	θ_{SY}
$\hat{\Pi}(1)$	$\|0\rangle$	$\|0\rangle$	π	π	π	π
$\hat{\Pi}(2)$	$\|0\rangle$	$\|1\rangle$	π	π	0	π
$\hat{\Pi}(3)$	$\|0\rangle$	$\|+\rangle$	π	π	$\pi/2$	0
$\hat{\Pi}(4)$	$\|0\rangle$	$\|-\rangle$	π	π	$\pi/2$	π
$\hat{\Pi}(5)$	$\|0\rangle$	$\|+i\rangle$	π	π	$\pi/2$	$\pi/2$
$\hat{\Pi}(6)$	$\|0\rangle$	$\|-i\rangle$	π	π	$\pi/2$	$3\pi/2$
$\hat{\Pi}(7)$	$\|1\rangle$	$\|0\rangle$	0	π	π	π
$\hat{\Pi}(8)$	$\|1\rangle$	$\|1\rangle$	0	π	0	π
$\hat{\Pi}(9)$	$\|1\rangle$	$\|+\rangle$	0	π	$\pi/2$	0
$\hat{\Pi}(10)$	$\|1\rangle$	$\|-\rangle$	0	π	$\pi/2$	π
$\hat{\Pi}(11)$	$\|1\rangle$	$\|+i\rangle$	0	π	$\pi/2$	$\pi/2$
$\hat{\Pi}(12)$	$\|1\rangle$	$\|-i\rangle$	0	π	$\pi/2$	$3\pi/2$
$\hat{\Pi}(13)$	$\|+\rangle$	$\|0\rangle$	$\pi/2$	0	π	π
$\hat{\Pi}(14)$	$\|+\rangle$	$\|1\rangle$	$\pi/2$	0	0	π
$\hat{\Pi}(15)$	$\|+\rangle$	$\|+\rangle$	$\pi/2$	0	$\pi/2$	0
$\hat{\Pi}(16)$	$\|+\rangle$	$\|-\rangle$	$\pi/2$	0	$\pi/2$	π
$\hat{\Pi}(17)$	$\|+\rangle$	$\|+i\rangle$	$\pi/2$	0	$\pi/2$	$\pi/2$
$\hat{\Pi}(18)$	$\|+\rangle$	$\|+i\rangle$	$\pi/2$	0	$\pi/2$	$3\pi/2$
$\hat{\Pi}(19)$	$\|-\rangle$	$\|0\rangle$	$\pi/2$	π	π	π
$\hat{\Pi}(20)$	$\|-\rangle$	$\|1\rangle$	$\pi/2$	π	0	π
$\hat{\Pi}(21)$	$\|-\rangle$	$\|+\rangle$	$\pi/2$	π	$\pi/2$	0
$\hat{\Pi}(22)$	$\|-\rangle$	$\|-\rangle$	$\pi/2$	π	$\pi/2$	π
$\hat{\Pi}(23)$	$\|-\rangle$	$\|+i\rangle$	$\pi/2$	π	$\pi/2$	$\pi/2$
$\hat{\Pi}(24)$	$\|-\rangle$	$\|-i\rangle$	$\pi/2$	π	$\pi/2$	$3\pi/2$
$\hat{\Pi}(25)$	$\|+i\rangle$	$\|0\rangle$	$\pi/2$	$\pi/2$	π	π
$\hat{\Pi}(26)$	$\|+i\rangle$	$\|1\rangle$	$\pi/2$	$\pi/2$	0	π
$\hat{\Pi}(27)$	$\|+i\rangle$	$\|+\rangle$	$\pi/2$	$\pi/2$	$\pi/2$	0
$\hat{\Pi}(28)$	$\|+i\rangle$	$\|-\rangle$	$\pi/2$	$\pi/2$	$\pi/2$	π
$\hat{\Pi}(29)$	$\|+i\rangle$	$\|+i\rangle$	$\pi/2$	$\pi/2$	$\pi/2$	$\pi/2$
$\hat{\Pi}(30)$	$\|+i\rangle$	$\|-i\rangle$	$\pi/2$	$\pi/2$	$\pi/2$	$3\pi/2$
$\hat{\Pi}(31)$	$\|-i\rangle$	$\|0\rangle$	$\pi/2$	$3\pi/2$	π	π
$\hat{\Pi}(32)$	$\|-i\rangle$	$\|1\rangle$	$\pi/2$	$3\pi/2$	0	π
$\hat{\Pi}(33)$	$\|-i\rangle$	$\|+\rangle$	$\pi/2$	$3\pi/2$	$\pi/2$	0
$\hat{\Pi}(34)$	$\|-i\rangle$	$\|-\rangle$	$\pi/2$	$3\pi/2$	$\pi/2$	π
$\hat{\Pi}(35)$	$\|-i\rangle$	$\|+i\rangle$	$\pi/2$	$3\pi/2$	$\pi/2$	$\pi/2$
$\hat{\Pi}(36)$	$\|-i\rangle$	$\|-i\rangle$	$\pi/2$	$3\pi/2$	$\pi/2$	$3\pi/2$

Supplementary Table 1 Over-complete set of measurements used in our experiment for the quantum state tomography.

Supplementary Discussion

On-chip state evolution

When a bright pump is presented at the input of our device, the on-chip quantum state of light evolves as follows. Starting with a coherent pump state, of $|\alpha|^2$ photons in the pump mode (mode b, referring to Supplementary Figure 6, and frequency p) b_p,

$$|\alpha\rangle_p \equiv \hat{D}(\alpha)|0\rangle_p \equiv \exp\left(\alpha b_p^\dagger - \alpha^* b_p\right)|0\rangle_p \tag{1}$$

we pass through the first coupler (leftmost of Supplementary Figure 6), whereby $b_p \to \sqrt{\eta} b_p - i\sqrt{1-\eta} d_p$, and $b_p^\dagger \to \sqrt{\eta} b_p^\dagger + i\sqrt{1-\eta} d_p^\dagger$, for a reflectivity $\eta \in [0,1]$. Thus,

$$\begin{aligned} |\alpha\rangle_p &\to e^{\alpha(\sqrt{\eta}b_p^\dagger + i\sqrt{1-\eta}d_p^\dagger) - \alpha^*(\sqrt{\eta}b_p - i\sqrt{1-\eta}d_p)}|0_b 0_d\rangle_p \\ &= e^{([\alpha\sqrt{\eta}]b_p^\dagger - [\alpha\sqrt{\eta}]^* b_p) + ([i\alpha\sqrt{1-\eta}]d_p^\dagger - [i\alpha\sqrt{1-\eta}]^* d_p)}|0_b 0_d\rangle_p \\ &= e^{[\alpha\sqrt{\eta}]b_p^\dagger - [\alpha\sqrt{\eta}]^* b_p} e^{[i\alpha\sqrt{1-\eta}]d_p^\dagger - [i\alpha\sqrt{1-\eta}]^* d_p}|0_b 0_d\rangle_p \\ &= |\alpha\sqrt{\eta}\rangle_{bp}|i\alpha\sqrt{1-\eta}\rangle_{dp} \end{aligned} \tag{2}$$

which is the quantum description of a laser splitting on a beamsplitter. Next, we will assume the pump passes through a random (but fixed) phase, which results in a θ_0 phase difference between modes b_p and d_p:

$$|\alpha\sqrt{\eta}\rangle_{bp}|i\alpha\sqrt{1-\eta}\rangle_{dp} \to |e^{i\theta_0}\alpha\sqrt{\eta}\rangle_{bp}|i\alpha\sqrt{1-\eta}\rangle_{dp}. \tag{3}$$

Next, the pump light impinges on the resonant SFWM sources. We know that

$$|\alpha\rangle \xrightarrow{\text{SFWM}} |\zeta\rangle_{II} = \sqrt{1-|\zeta|^2}\sum_{n=0}^{\infty}(-\zeta)^n|n_s n_i\rangle \tag{4}$$

where $\zeta \equiv ie^{i\arg(t\gamma\alpha^2)}\tanh|t\gamma\alpha^2|$ which for small $t\gamma\alpha^2$ becomes $\zeta \sim \xi \equiv it\gamma\alpha^2$, so in the *weak-pump* regime:

$$|\alpha\rangle \xrightarrow{\text{SFWM}} |0_s 0_i\rangle - \xi\,|1_s 1_i\rangle + \mathcal{O}\left(\xi^2\right). \tag{5}$$

The state resulting from SFWM pumped by a weak coherent field (5), then, is normalised in the limit of zero squeezing ($\xi \to 0$), and almost always gives only vacuum. Occasionally, though, a single pair is produced, and it is these events that we will be focussing on in this analysis. Returning to our evolving photonic state, we can now apply the weak-pump SFWM:

$$\begin{aligned} |e^{i\theta_0}\alpha\sqrt{\eta}\rangle_{bp}|i\alpha\sqrt{1-\eta}\rangle_{dp} &\to \left(|0_s 0_i\rangle_b - \xi_b\,|1_s 1_i\rangle_b\right) \otimes \left(|0_s 0_i\rangle_d - \xi_d\,|1_s 1_i\rangle_d\right) \\ &= |\text{vac}\rangle - \xi_d|0_s 0_i\rangle_b|1_s 1_i\rangle_d - \xi_b|1_s 1_i\rangle_b|0_s 0_i\rangle_d + \mathcal{O}(\xi_b\xi_d)\,. \end{aligned} \tag{6}$$

Here, we have defined separate squeeze parameters for the top and bottom sources as $\xi_b \equiv it\gamma_b e^{i2\theta_0}\alpha^2\eta$ and $\xi_d \equiv -t\gamma_d\alpha^2(1-\eta)$, respectively.

Notice at this point that (6) is merely a separable product of squeezed states. Each of those squeezed states is not individually separable, but this non-separability is not the source of entanglement in this experiment—it merely provides the correlations, which are rooted in energy conservation. It would be surprising if any two naturally probabilistic sources, anywhere in the universe, pumped coherently, were intrinsically entangled. This is also not the case here. The Bell-type entanglement generated in this experiment—and, indeed, in any down-conversion-based entanglement scheme—arises from the next step, where we post-select away the vacuum component of (6), and assume that the probability of multi-pair events is negligible.

Post-selecting on coincidences in our detectors (i.e. we cannot measure vacuum), and again assuming that $\xi^2 \ll \xi$, we obtain the following entangled state, after the sources:

$$\xi_d|0_s 0_i\rangle_b|1_s 1_i\rangle_d + \xi_b|1_s 1_i\rangle_b|0_s 0_i\rangle_d. \tag{7}$$

This light then is divided by frequency inside the on-chip filters, which, referring again to Supplementary Figure 6, take

$$\begin{aligned} b_s &\to a_s \quad & d_s &\to c_s \\ b_i &\to b_i \quad & d_i &\to d_i \end{aligned}$$

by which, relabelling

$$\begin{aligned} &\xi_d|0_s 0_i\rangle_b|1_s 1_i\rangle_d + \xi_b|1_s 1_i\rangle_b|0_s 0_i\rangle_d \\ &\to \xi_d|0\rangle_{as}|0\rangle_{bi}|1\rangle_{cs}|1\rangle_{di} + \xi_b|1\rangle_{as}|1\rangle_{bi}|0\rangle_{cs}|0\rangle_{di} \end{aligned} \tag{8}$$

now we apply the crosser at the centre of the scheme, to relabel $b_i \to c_i$ and $c_s \to b_s$, and we obtain the state of interest, which we will name $|\phi^\pm\rangle$:

$$|\phi^\pm\rangle \equiv \xi_b|1\rangle_{as}|0\rangle_{bs}|1\rangle_{ci}|0\rangle_{di} + \xi_d|0\rangle_{as}|1\rangle_{bs}|0\rangle_{ci}|1\rangle_{di} \tag{9}$$

Let's take a moment to reflect on the properties of $|\phi^{\pm}\rangle$. First, it doesn't seem normalised, since we have placed no restriction on the magnitudes of ξ_b or ξ_d. This is to be expected, since any extra normalisation is taken up by the (very large) vacuum and the (very small) multi-pair contributions, which we have omitted, as discussed. To normalise $|\phi^{\pm}\rangle$, we define the photon flux balance between the two sources β. It ranges from 0 to 1 when the top or bottom sources dominate, respectively, and is 1/2 when they are balanced.

$$\beta \equiv \frac{|\xi_b|^2}{|\xi_b|^2+|\xi_d|^2} = 1-\frac{|\xi_d|^2}{|\xi_b|^2+|\xi_d|^2} = \frac{\gamma_b^2\eta^2}{\gamma_b^2\eta^2+\gamma_d^2(1-\eta)^2} \tag{10}$$

Using this definition for β, and lumping the phases onto the bottom mode in a total internal phase Θ, we can rewrite the squeeze parameters in a clean and normalised way:

$$\begin{gathered}\Theta \equiv \pi/2 - 2\theta_0 \\ \xi_b \rightarrow \sqrt{\beta} \qquad \xi_d \rightarrow e^{i\Theta}\sqrt{1-\beta}\end{gathered} \tag{11}$$

and obtain a new form of $|\phi^{\pm}\rangle$

$$|\phi^{\pm}\rangle = \sqrt{\beta}|1\rangle_{as}|0\rangle_{bs}|1\rangle_{ci}|0\rangle_{di} + e^{i\Theta}\sqrt{1-\beta}|0\rangle_{as}|1\rangle_{bs}|0\rangle_{ci}|1\rangle_{di} \tag{12}$$

Second, the state contains four modes with two orthogonal frequencies. Obviously, photons of different frequencies don't interfere, and so only modes a and b or c and d can. Third, $|\phi^{\pm}\rangle$ has a variable amount of entanglement, depending on the ratio of the two squeeze parameters. If β is either 0 or 1 (ξ_b or ξ_d is zero), $|\phi^{\pm}\rangle$ becomes unentangled, and takes the form $|\phi^{\pm}\rangle = |0101\rangle$ or $|\phi^{\pm}\rangle = |1010\rangle$, respectively. Conversely, if $\beta = 1/2$, then $|\phi^{\pm}\rangle \equiv |\phi^{\pm}_{\text{max}}\rangle$ is maximally entangled. In fact, if we adopt the dual-rail qubit scheme, and form qubits from (adjacent) modes of identical frequency, we can re-write $|\phi^{\pm}_{\text{max}}\rangle$ in the qubit basis,

$$|\phi^{\pm}_{\text{max}}\rangle = \frac{|\mathbf{00}\rangle + e^{i\Theta}|\mathbf{11}\rangle}{\sqrt{2}} \tag{13}$$

and identify $|\phi^{\pm}_{\text{max}}\rangle$ with the maximally entangled two-qubit Bell states $|\Phi^{\pm}\rangle$ for $\Theta = \{0, \pi\}$.

In the above derivation, we assumed that photon pairs were produced into a strictly two-mode squeezed vacuum (i.e. were frequency-separable) and shared identical spectral properties. Here, we'll remove these assumptions, and treat the case of general spectral properties.

We'll expand the source efficiencies ξ to include a joint spectral component, which governs the biphotonic spectral dependence. The joint spectra are normalised with respect to the signal and idler frequencies ν_s and ν_i, and the *overlap* σ quantifies the similarity between the two spectra.

$$\begin{gathered}\sum_s\sum_i |J_b(\nu_s,\nu_i)|^2 = \sum_s\sum_i |J_d(\nu_s,\nu_i)|^2 = 1 \\ \sigma \equiv \sum_s\sum_i J_b(\nu_s,\nu_i)\cdot J_d^*(\nu_s,\nu_i)\end{gathered} \tag{14}$$

Thus, (9) becomes

$$|\phi^{\pm}\rangle \equiv \sum_s\sum_i \Big(\xi_b J_b(\nu_s,\nu_i)|1010\rangle_{s,i} + \xi_d J_d(\nu_s,\nu_i)|0101\rangle_{s,i}\Big) \tag{15}$$

Here there is an intricacy, in that we can't measure the difference between the various $\{\nu_s\}$s and $\{\nu_i\}$s, so we must trace over them. This means we must add up all the probabilities of the indistinguishable events, and implies that we must collapse our basis of all frequencies down into one which contains only spatial and $s \leftrightarrow i$ information. In other words, we can take $\sum|f|^2$ rather than $|\sum f|^2$. Thus (9) can be written in the form of a density matrix:

$$\begin{aligned}\hat{\rho} = |\phi^{\pm}\rangle\langle\phi^{\pm}| = &|1010\rangle\langle 1010|\cdot|\xi_b|^2\sum_s\sum_i J_b(\nu_s,\nu_i)J_b^*(\nu_s,\nu_i) \\ &+ |0101\rangle\langle 0101|\cdot|\xi_d|^2\sum_s\sum_i J_d(\nu_s,\nu_i)J_d^*(\nu_s,\nu_i) \\ &+ |1010\rangle\langle 0101|\cdot\xi_b\xi_d^*\sum_s\sum_i J_b(\nu_s,\nu_i)J_d^*(\nu_s,\nu_i) \\ &+ |0101\rangle\langle 1010|\cdot\xi_b^*\xi_d\sum_s\sum_i J_d(\nu_s,\nu_i)J_b^*(\nu_s,\nu_i)\end{aligned} \tag{16}$$

This can now be expressed using the definitions of (14) and (11) in terms of the overlap σ, balance β, and lumped internal phase Θ, and converted to the qubit basis, such that

$$\begin{aligned}\hat{\rho} = \;&|00\rangle\langle 00|\cdot|\xi_b|^2 &&= |00\rangle\langle 00|\cdot\beta \\ &+|11\rangle\langle 11|\cdot|\xi_d|^2 &&+|11\rangle\langle 11|\cdot(1-\beta) \\ &+|00\rangle\langle 11|\cdot\xi_b\xi_d^*\cdot\sigma &&+|00\rangle\langle 11|\cdot e^{-i\Theta}\sqrt{\beta}\sqrt{1-\beta}\cdot\sigma \\ &+|11\rangle\langle 00|\cdot\xi_b^*\xi_d\cdot\sigma^* &&+|11\rangle\langle 00|\cdot e^{+i\Theta}\sqrt{\beta}\sqrt{1-\beta}\cdot\sigma^*\end{aligned} \tag{17}$$

The last, qubit-encoded line of (17) tells us the density matrix we should expect, for source (or WDM) resonances with an imperfect overlap σ. A nice way to look at this result is in terms of the perfect, pure response, and the perfectly mixed response. In the pure case, there are maximal coherent side lobes, since the state is perfectly coherent, while in the mixed case, the energy correlations are still present, but the coherence is entirely lost. This latter case is like flipping two coins which have been glued together, while the former case represents true quantum entanglement. The parameter σ smoothly moves the output state between these two regimes.

$$\hat{\rho} = \sigma\hat{\rho}_{\text{pure}} + (1-\sigma)\hat{\rho}_{\text{mixed}} \tag{18}$$

Supplementary Methods

Overlap, fringe visibility, and multi-pair events

In Fig. 2b of the main text, we show the visibility of reverse-HOM-type fringes between our two microring sources, in the configuration of Supplementary Reference 1. To convert these visibility data into overlap data (σ), some additional modelling was needed. We replaced (4) for one of the sources with a new definition, which involves a coherent superposition of two frequencies: the original s and i and two new orthogonal frequencies, labelled s' and i'.

$$|\beta\rangle \xrightarrow{\text{SFWM}} |\zeta\rangle'_{II} \equiv \sqrt{1-|\zeta|^2}\sum_{n=0}^{\infty}(-\zeta)^n\left(\sigma|n_s n_i\rangle + \sqrt{1-\sigma^2}|n_{s'}n_{i'}\rangle\right) \tag{19}$$

In this way, when $\sigma = 1$ we recover the original expression, but, when $\sigma = 0$, these new-frequency photons (at s' and i') will not interfere with those from the other source (still at the original frequencies s and i). Here, we implicitly assume that each source produces separable photon pairs—further modelling indicates only a small deviation from these values in the case of frequency-correlated pairs.

The maximum achievable visibility is plotted against the pair generation probability ($\approx |\zeta|^2$ for each source) in Supplementary Figure 3, together with our measured result. It shows that the spectral indistinguishability of our sources is very close to unity, when considering the pair generation probability of 0.075 used here. When the measured imbalance of our sources is included, the difference becomes even narrower. Finally, we can compute the relationship between spectral overlap and visibility, while accounting for multi-pair events, as shown in Supplementary Figure 4. From this, and including the source balance $\beta = 0.43$ we can estimate an experimental overlap of $\sigma = 0.99 \pm 0.08$. The overlap uncertainty is computed as $(\mathrm{d}V/\mathrm{d}\sigma)^{-1} \times \delta V$, where δV is the uncertainty in the visibility (0.021), and the derivative is evaluated numerically at the measured value, $V = 0.958$.

The Bell-CHSH test

A well known test of quantum mechanical non-locality, as well as a very strict indicator of the entanglement present in a quantum state (Supplementary Reference 2), is based on the formulation of Bell's original inequality due to Clauser, Horne, Shimony and Holt (CHSH). In this test, a parameter S is evaluated based on a series of measurements on the state in question:

$$S \equiv |E(\mathbf{a},\mathbf{b}) + E(\mathbf{a}',\mathbf{b}) + E(\mathbf{a},\mathbf{b}') - E(\mathbf{a}',\mathbf{b}')| \leq 2. \tag{20}$$

where $\mathbf{a}$, $\mathbf{a}'$, $\mathbf{b}$, and $\mathbf{b}'$ are measurement operators chosen independently by two observers (Alice and Bob), each performing measurements on one of the two qubits. If the state under test obeys a local hidden variable theory, the inequality $-2 \leq S \leq 2$ is satisfied, while if the inequality is violated the state is entangled and it can reach a maximum value of $S < 2\sqrt{2}$ (Supplementary Reference 3,4). As shown in Supplementary Reference 2, the amount of violation is a very strict estimation of the entanglement in the state. We estimated S for each of the several reconstructed density matrices of the states produced by the device.

To measure the CHSH parameter directly, we configured the signal and idler $\hat{R}_y$ rotations to mix the two modes of each qubit (i.e. $\hat{R}_y(\theta_{\text{SY}}) = \hat{R}_y(\theta_{\text{IY}}) = \hat{H}$, with $\theta_{\text{SY}} = \theta_{\text{IY}} = \pi/2$), then rotated both θ_{SZ} and θ_{IZ}, to observe coincidence fringes with an entangled phase equal to $\theta_{\text{SZ}} + \theta_{\text{IZ}}$. We show below how S can be extracted from the visibility of these fringes.

First, we must obtain the expectation values of the projections along $\mathbf{a}$ and $\mathbf{b}$ from our density matrix $\hat{\rho}$. A generalised Pauli matrix, which decomposes a vector $\mathbf{a}$ on the Bloch Sphere into it's component $\hat{\sigma}_x$, $\hat{\sigma}_y$, and $\hat{\sigma}_z$ operators, written in spherical coordinates θ and ϕ, is given by

$$\hat{\sigma}_{\text{gen}}(\mathbf{a}) \equiv \mathbf{a}\cdot\begin{pmatrix}\hat{\sigma}_x\\ \hat{\sigma}_y\\ \hat{\sigma}_z\end{pmatrix} = \hat{\sigma}_x\sin(\theta)\cos(\phi) + \hat{\sigma}_y\sin(\theta)\sin(\phi) + \hat{\sigma}_z\cos(\theta). \tag{21}$$

The operator $\hat{\sigma}_{\text{gen}}(\mathbf{a})$ is Hermitian, and represents a single-qubit observable which projects onto $\mathbf{a}$. Knowing this, we can write the vectors $\mathbf{a}$ and $\mathbf{b}$ of (20) as expectation values of the observables $\hat{\sigma}_{\text{gen}}$ for each of the two qubits a and b, and re-cast $E(\mathbf{a},\mathbf{b})$ in terms of the angles θ and ϕ:

$$\begin{aligned} E(\mathbf{a},\mathbf{b}) &= \langle\hat{\sigma}_{\text{gen}}(\mathbf{a})\otimes\hat{\sigma}_{\text{gen}}(\mathbf{b})\rangle \\ &= \begin{cases}\langle\psi|\hat{\sigma}_{\text{gen}}(\mathbf{a})\otimes\hat{\sigma}_{\text{gen}}(\mathbf{b})|\psi\rangle & \text{pure state } |\psi\rangle \\ \mathrm{Tr}\left[\hat{\rho}\cdot\hat{\sigma}_{\text{gen}}(\mathbf{a})\otimes\hat{\sigma}_{\text{gen}}(\mathbf{b})\right] & \text{general state } \hat{\rho}\end{cases} \end{aligned} \tag{22}$$

$$E(\mathbf{a},\mathbf{b}) \to E(\theta_a,\phi_a,\theta_b,\phi_b) = \langle \hat{\sigma}_{\text{gen}}(\theta_a,\phi_a) \otimes \hat{\sigma}_{\text{gen}}(\theta_b,\phi_b) \rangle \tag{23}$$

The degree of violation depends strongly on one's choice of measurement bases. For our target state $|00\rangle + e^{-i2\theta_0}|11\rangle$ the maximum violation occurs at

$$\begin{aligned} \theta_a &= 0 & \phi_a &= 0 \\ \theta_{a'} &= \pi/2 & \phi_{a'} &= 0 \\ \theta_b &= 3\pi/4 & \phi_b &= \pi + 2\theta_0 \\ \theta_{b'} &= \pi/4 & \phi_{b'} &= \pi + 2\theta_0 \end{aligned}$$

With these rotations, we can apply the test to the state (17) and obtain the following expression for the CHSH parameter

$$S = \sqrt{2}\left(1 + 2\sigma\sqrt{\beta(1-\beta)}\right) \tag{24}$$

which, as expected, gives a maximal violation of $2\sqrt{2}$ when the overlap ($\sigma = 1$) and balance ($\beta = 1/2$) are optimised to produce a maximally entangled state. This dependence of the Bell-CHSH parameter on the source overlap and brightness balance is plotted in Fig. 3b of the main text.

To predict the effect of the overlap (σ) and balance (β) on the Bell fringe visibility (Fig. 3b in the main text), we start with the expression for the partially mixed state given by (17), and apply the two projectors $\hat{U}_{\text{S}} \equiv \hat{R}_z(\theta_{\text{SZ}})\hat{R}_y(\pi/2)$ and $\hat{U}_{\text{I}} \equiv \hat{R}_z(\theta_{\text{IZ}})\hat{R}_y(\pi/2)$

$$\hat{U}_{\text{S}} = \frac{1}{\sqrt{2}}\begin{pmatrix} e^{i\theta_{\text{SZ}}} & 1 \\ e^{i\theta_{\text{SZ}}} & -1 \end{pmatrix} \qquad \hat{U}_{\text{I}} = \frac{1}{\sqrt{2}}\begin{pmatrix} e^{i\theta_{\text{IZ}}} & 1 \\ e^{i\theta_{\text{IZ}}} & -1 \end{pmatrix} \tag{25}$$

$$\hat{U} = \hat{U}_{\text{S}} \otimes \hat{U}_{\text{I}} = \frac{1}{2}\begin{pmatrix} e^{i(\theta_{\text{IZ}}+\theta_{\text{SZ}})} & e^{i\theta_{\text{SZ}}} & e^{i\theta_{\text{IZ}}} & 1 \\ e^{i(\theta_{\text{IZ}}+\theta_{\text{SZ}})} & e^{i\theta_{\text{SZ}}} & e^{i\theta_{\text{IZ}}} & 1 \\ e^{i(\theta_{\text{IZ}}+\theta_{\text{SZ}})} & e^{i\theta_{\text{SZ}}} & e^{i\theta_{\text{IZ}}} & 1 \\ e^{i(\theta_{\text{IZ}}+\theta_{\text{SZ}})} & e^{i\theta_{\text{SZ}}} & e^{i\theta_{\text{IZ}}} & 1 \end{pmatrix} \qquad \hat{\rho} \to \hat{U}\hat{\rho}\hat{U}^{\dagger} \tag{26}$$

For a Bell fringe projecting on $|\mathbf{00}\rangle$ we expect oscillations coupled between the signal and idler Z phases, and the lumped internal phase of the state, with a visibility dependent on the overlap and balance, and indeed this is what we find. Looking at the **00** qubit state at the output, we predict fringes of the form

$$P_{\mathbf{00}} = \frac{1 + 2\sigma\sqrt{\beta(1-\beta)}\cos(\Theta - \theta_{\text{SZ}} - \theta_{\text{IZ}})}{4} \tag{27}$$

Explicitly, the $P_{\mathbf{00}}$ fringe visibility has the dependency

$$V = \frac{4\sigma\sqrt{\beta(1-\beta)}}{1 + 2\sigma\sqrt{\beta(1-\beta)}} \quad \to \quad 2\sigma\sqrt{\beta(1-\beta)} = \frac{V}{2-V} \tag{28}$$

Comparing this result with (24) we see immediately that the fringe visibility and the CHSH S parameter have a one-to-one correspondence:

$$S = \sqrt{2}\left(1 + \frac{V}{2-V}\right) \quad \leftrightarrow \quad V = 2 - \frac{2\sqrt{2}}{S} \tag{29}$$

Equation (29) tells us that if we can obtain fringes with a visibility above $2 - \sqrt{2} \approx 59\%$, then the Bell-CHSH inequality will be violated. Measured fringes are plotted in Fig. 3b of the main text, showing visibilities between 93% and 96%. Finally, according to (29), this state has an S parameter in the range from 2.64 and 2.71.

Quantum state tomography

In order to reconstruct the state, we performed quantum state tomography using constrained least square (CLS) estimation and an over-complete set of measurements. By choosing an over-complete set of measurements, we increase the accuracy of the tomographic reconstruction, as shown in Supplementary Reference **5**.

We calibrated the analysis interferometers, as described in the Methods, in order to identify the voltages necessary to set the SU(2) rotations required to produce an informationally complete set of projective measurements. An informationally complete set of measurements must consist of at least as many measurements as there are degrees of freedom (parameters) in the state. So, for example, in the single qubit case, there are three unknown parameters describing the 2×2 density matrix, plus one unknown parameter necessary for the normalisation that will depend on several experimental parameters, like brightness of the source, losses and integration time (Supplementary Reference **6**). Therefore a total of four parameters and an appropriate set of projectors will be composed by, at least, four elements, for example:

$$\{|0\rangle\langle 0|,\ |1\rangle\langle 1|,\ |+\rangle\langle +|,\ |+i\rangle\langle +i|\}. \tag{30}$$

Meanwhile an over-complete set of measurements is composed of a larger number of elements, for example six:

$$\{|0\rangle\langle 0|,\ |1\rangle\langle 1|,\ |+\rangle\langle +|,\ |-\rangle\langle -|,\ |+i\rangle\langle +i|,\ |-i\rangle\langle -i|\}. \tag{31}$$

We must keep in mind, however, that in order to properly normalise the additional measurements, we must perform measurements which sample all possible single-measurement outcomes (for example, 00, 01, 10, and 11 must be measured together). We can describe the set of measurements as projectors onto certain states ($|0\rangle$, $|1\rangle$, $|+\rangle = (|0\rangle + |1\rangle)/\sqrt{2}$, $|-\rangle = (|0\rangle - |1\rangle)/\sqrt{2}$, $|+i\rangle = (|0\rangle + i|1\rangle)/\sqrt{2}$, and $|-i\rangle = (|0\rangle - i|1\rangle)/\sqrt{2}$).

In the 2 qubit case the density operator is instead a 4×4 matrix and so the total number of parameters is $15 + 1 = 16$. Consequently the smallest informationally complete set of projectors has 16 elements. For example one possible choice is to use the single-qubit informationally complete set of projectors (30) for each of the two qubits and perform all the possible combinations. One possible over-complete set of measurements is obtained by using all possible combinations of the single qubit over-complete measurement set in (31). This is the measurement set used in this experiment, shown explicitly in Supplementary Table 1.

For each of the $6 \times 6 = 36$ measurements, see Table 1, we collected coincidence counts. The experimental data set obtained using these projectors is composed of 36 coincidence counts which we denote by a single index for simplicity: $C_1, \ldots, C_i, \ldots, C_{36}$. This dataset was then used by the search algorithm for the estimation of the density matrix. The density operator $\hat{\rho}$ of a 2 qubits state can be represented by an Hermitian, positive definite matrix. For this reason, it is possible to use the Cholesky decomposition and express the density matrix as product of a lower triangular matrix $\hat{\tau}$ and its Hermitian conjugate $\hat{\tau}^\dagger$:

$$\hat{\rho} = \hat{\tau} \cdot \hat{\tau}^\dagger \tag{32}$$

where:

$$\hat{\tau} = \begin{pmatrix} t_1 & 0 & 0 & 0 \\ t_5 + it_6 & t_2 & 0 & 0 \\ t_{11} + it_{12} & t_7 + it_8 & t_3 & 0 \\ t_{15} + it_{16} & t_{13} + it_{14} & t_9 + it_{10} & t_4 \end{pmatrix} \tag{33}$$

and where $t_1, t_2, ..., t_{16}$ are real numbers.

In order to be physical, a density matrix, must be Hermitian, positive definite, and have a unitary trace. We must impose these conditions on candidate density matrices during the estimation process. The search algorithm used for the state estimation can be schematically summarised by the following steps:

- Get a new trial list of the $\{t_i\}$ parameters from (32), which describes a physical density matrix.
- Generate the density matrix at the nth step, $\hat{\rho}(t_1, \ldots t_{16})_n$, expressed as a function of the 16 parameters in $\{t_i\}$.
- Evaluate an error function, to estimate the distance between the generated density matrix $\hat{\rho}_n$ and the (inaccessible) true density matrix $\hat{\rho}_{\text{ex}}$ which is generating the experimental data set.

In searching for the $\hat{\tau}$ matrix (eq. 32), the Hermitian and positive definiteness conditions are automatically imposed. We also impose normalisation at each step using the trace.

We used a simulated annealing algorithm to find the $\{t_i\}$. As shown in the main text Methods, the problem can be defined as a search for the density matrix which minimises the error quantity, Δ. The algorithm searches the 16-dimensional space (the space of the parameters $t_1, \ldots t_{16}$) to minimise Δ. We used the constrained least squares estimator, which requires the minimisation of:

$$\Delta = \sum_i \left| P_{\text{ex}}(i) - P_{\hat{\rho}}(i) \right|^2 \tag{34}$$

Where $P_{\hat{\rho}}(i)$ is the theoretical probability associated with the ith projector for the estimated density matrix, and is defined as:

$$P_{\hat{\rho}}(i) = \text{Tr}\left\{\hat{\rho} \cdot \hat{\Pi}(i)\right\} \tag{35}$$

And $P_{\text{ex}}(i)$ is the experimentally obtained probability for the same projector. In this experiment, as explained previously, the data collected consist of frequencies—coincidence counts in a fixed time interval. We extrapolate the probabilities from the coincidences. To normalise them, we define the experimental probabilities in the following way:

$$P_{\text{ex}}(i) = \frac{CC_{\text{ex}}(i) \times \sum\limits_{j=1}^{N_{\text{tot}}} P_{\hat{\rho}}(j)}{\sum\limits_{j=1}^{N_{tot}} CC_{\text{ex}}(j)} \tag{36}$$

Where $CC_{\text{ex}}(j)$ are the coincidence counts recorded for the jth projector and N_{tot} is the total number of projectors, in our case 36.

Joint spectral density simulation

The main steps required to derive the joint spectral density are detailed in Supplementary Reference 7. Here we summarise these steps and state the expression used for our simulations. We start with the four-wave mixing Hamiltonian written in terms of the eigenmode of the ring resonator structure we consider

$$\hat{H}_{NL} = -\int dk_1 dk_2 dk_3 dk_4 f(k_1) e^{ik_1 L} a^\dagger_{k_1} f(k_2) e^{ik_2 L} a^\dagger_{k_2} f^\star(k_3) e^{-ik_3 L} a_{k_3} f^\star(k_4) e^{-ik_4 L} a_{k_4} + H.c. \tag{37}$$

where $f(k) = \frac{i\kappa}{1-\sigma e^{ikL}}$ is the field enhancement in the ring resonator (κ is the bus coupling and $\sigma = \tau\sqrt{1-\kappa^2}$ with τ being the round trip transmission in the ring cavity, L is the length of the ring)

Seeding the process with a coherent state $|\alpha\rangle = e^{\alpha \int dk \Phi_p(k) a^\dagger_k}$, and expanding the unitary evolution to first order, one can demonstrate that the bi-photon state generated in the ring resonator has the following form

$$|\Psi_{out}\rangle = \left[1 + \frac{i\beta}{\sqrt{2}} \iint dk_1 dk_2 \Phi(k_1, k_2) a^\dagger_{k_1} a^\dagger_{k_2}\right] |\text{vac}\rangle \tag{38}$$

where β is the probability of getting a photon pair per pulse and Φ is the normalised joint probability amplitude of emitting two photons with given energies ω_1 and ω_2 related to their respective wavenumbers k_1 and k_2. The shape of Φ is governed by the shape of the pump spectrum Φ_p, the ring resonator source spectral response f, the energy conservation and the phase mismatch $\Delta k = k_1 + k_2 - k_3 - k_4$.

$$\Phi(k_1, k_2) \propto \iint dk_3 dk_4 \Phi_p(k_3) \Phi_p(k_4) \text{sinc}\left(\frac{\Delta k L}{2}\right) f(k_1) f(k_2) f^\star(k_3) f^\star(k_4) \delta(\omega_1 + \omega_2 - \omega_3 - \omega_4) \tag{39}$$

We define the normalised joint probability amplitude function in the frequency space $\tilde{\Phi}(\omega_1, \omega_2) \equiv v_g \Phi(k_1, k_2)$ with v_g being the group velocity in the medium considered. Then using the change of variable $dk \to \frac{1}{v_g} d\omega$

$$\tilde{\Phi}(\omega_1, \omega_2) \propto \int d\omega_3 \Phi_p\left(k(\omega_3)\right) \Phi_p\left(k(\omega_1 + \omega_2 - \omega_3)\right) \text{sinc}\left(\frac{\Delta k L}{2}\right) f(k(\omega_1)) f(k(\omega_2)) f^\star(k(\omega_3)) f^\star(k(\omega_1 + \omega_2 - \omega_3)) \tag{40}$$

Finally, the simulation is performed by numerically evaluating $\tilde{\Phi}(\omega_i, \omega_s)$ for each signal and idler frequency ω_i, ω_s.

Supplementary References

[1] J. W. Silverstone, D. Bonneau, K. Ohira, N. Suzuki, H. Yoshida, N. Iizuka, M. Ezaki, C. M. Natarajan, M. G. Tanner, R. H. Hadfield, V. Zwiller, G. D. Marshall, J. G. Rarity, J. L. O'Brien, and M. G. Thompson. On-chip quantum interference between silicon photon-pair sources. *Nature Photon.*, 8(2):104–108, February 2014.

[2] K. Bartkiewicz, B. Horst, K. Lemr, and A. Miranowicz. Entanglement estimation from bell inequality violation. *Phys. Rev. A*, 88:052105, November 2013.

[3] A. Aspect, P. Grangier, and G. Roger. Experimental realization of einstein-podolsky-rosen-bohm gedankenexperiment: A new violation of bell's inequalities. *Phys. Rev. Lett.*, 49:91–94, July 1982.

[4] J. F. Clauser, M. A. Horne, A. Shimony, and R. A. Holt. Proposed experiment to test local hidden-variable theories. *Phys. Rev. Lett.*, 23:880–884, October 1969.

[5] M. D. de Burgh, N. K. Langford, A. C. Doherty, and A. Gilchrist. Choice of measurement sets in qubit tomography. *Phys. Rev. A*, 78:052122, November 2008.

[6] D. F. V. James, P. G. Kwiat, W. J. Munro, and A. G. White. Measurement of qubits. *Phys. Rev. A*, 64:052312, October 2001.

[7] M. Liscidini and J. E. Sipe. Asymptotic fields for a Hamiltonian treatment of nonlinear electromagnetic phenomena. *Phys. Rev. A*, 85(1):013833, January 2012.
